# Neuroimaging in mental health care: voices in translation

**DOI:** 10.3389/fnhum.2012.00293

**Published:** 2012-10-22

**Authors:** Emily L. Borgelt, Daniel Z. Buchman, Judy Illes

**Affiliations:** National Core for Neuroethics, Department of Neurology, University of British ColumbiaVancouver, BC, Canada

**Keywords:** neuroethics, bioethics, qualitative research, neuroimaging, psychiatry

## Abstract

Images of brain function, popularly called “neuroimages,” have become a mainstay of contemporary communication about neuroscience and mental health. Paralleling media coverage of neuroimaging research and the high visibility of clinics selling scans is pressure from sponsors to move basic research about brain function along the translational pathway. Indeed, neuroimaging may offer benefits to mental health care: early or tailored intervention, opportunities for education and planning, and access to resources afforded by objectification of disorder. However, risks of premature technology transfer, such as misinterpretation, misrepresentation, and increased stigmatization, could compromise patient care. The insights of stakeholder groups about neuroimaging for mental health care are a largely untapped resource of information and guidance for translational efforts. We argue that the insights of key stakeholders—including researchers, healthcare providers, patients, and families—have an essential role to play upstream in professional, critical, and ethical discourse surrounding neuroimaging in mental health. Here we integrate previously orthogonal lines of inquiry involving stakeholder research to describe the translational landscape as well as challenges on its horizon.

## Introduction

Images of brain function have become a mainstay of contemporary communication about neuroscience and mental health. Paralleling media coverage of neuroimaging research and the proliferation of highly publicized clinics in the United States, Canada, and Japan that sell brain scans for mental health assessments, is pressure from sponsors to move basic research about brain function along the translational pathway. Indeed, neuroimaging may offer potential benefits to mental health care: early or tailored intervention, opportunities for education and planning, and access to resources afforded by image representation of disease processes. However, premature or inappropriate technology transfer poses many potential risks, such as misrepresentation or misunderstanding of results that can compromise patient care. Some scientists and ethicists also caution against risks of increased social distance, stigma, and marginalization. To date, discussion about translational efforts largely misses a prime opportunity to inform professional debates, risk-benefit assessments, and policy developments with direct knowledge of how end-user stakeholders could be affected. Here, we synthesize known perspectives about research on clinical neuroimaging in mental health care, and advance an imperative to shift stakeholder inquiry from back stage murmurs to front stage voices. We note that our discussion focuses on functional neuroimaging modalities, and we use the term neuroimage to refer to images of brain function.

## Perspectives on a “brain frame” for mental health

Brain disease models of mental health and illness dominate current Western psychiatric theory and practice, displacing earlier notions of psychopathology as disorders of psyche, “maladies of the soul,” or moral shortcomings. Support for this shift toward a neuroscientific epistemology has been demonstrated by initiatives in the United States such as the 1990 launch of the Decade of the Brain by the Library of Congress and National Institute of Mental Health (NIMH), a multi-institutional effort to promote brain research (Project on the Decade of the Brain, 2000), and the codification of a goal to “*accelerate the pace of new discoveries by fostering research that translates findings from basic science into new treatments addressing fundamental mechanisms that cut across current diagnostic categories*” by the Research Domain Criteria (RDoC) program of the NIMH (Insel et al., 2010). Prominent psychiatrists have endorsed a brain disease model of mental illness as a reconceptualization necessary to “*transform the practice of psychiatry*” with the legitimizing force of neuroscience (Insel and Wang, 2010).

Given the prevailing brain disease model, it is not surprising that mental health research has embraced neuroimaging technologies such as positron emission tomography (PET), single photon emission computed tomography (SPECT) and, most recently, functional magnetic resonance imaging (fMRI) as instrumental to the identification of neural processes underlying mental health diagnoses (Agarwal et al., 2010). The hope is that neuroimaging research will contribute to evidence-based practice, and lead to refined diagnostics and targeted treatment directed by neurobiology (Lennox, 2009; Linden and Fallgatter, 2009; Insel and Wang, 2010). Indeed, the promises of an anticipated translation of neuroimaging—potentially combined with genetic testing (Linden and Thome, 2011)—to mainstream psychiatric clinical care in the future are significant. Already some private clinics have capitalized on the therapeutic promise of neuroimaging by offering brain scans as part of mental health evaluation—a service whose high demand is evidenced by rapid expansion and lengthy wait lists (Cyranoski, 2011; Amen Clinics Home Page, 2012). This industry thrives despite a dearth of support for routine clinical use of neuroimaging by the broader psychiatric community (American Psychiatric Association, 2005; Adinoff and Devous, 2010a,b).

Despite the breadth of neuroimaging research on mental health disorders, missing from discussions about the anticipated integration of neuroimaging in routine mental health care are the voices of stakeholders. While everyone in society may be affected to varying degrees by the outcome of medical technologies, here we use the term stakeholder more narrowly to refer to individuals who have a vested interest in current and future applications of neuroimaging in mental health care or who are the intended users or beneficiaries of the technology. Stakeholders may include neuroimaging and mental health researchers, providers at all levels of care, individuals living with mental health disorders as well as their employers, caregivers, and loved ones. If neuroimaging technology might promote both physical and mental well-being as proponents suggest, should not stakeholder priorities, experiences, concerns, and expectations be known and integrated into translational efforts? We think the answer is yes. As neuroimaging technology advances along its projected trajectory toward the clinic and scholars engage in ethics recommendations and policy development, stakeholder perspectives have an essential role to play in informing the ethical advancement of neurotechnologies and patient care. In 2010, anthropologist and ethnographer on issues in mental health Emily Martin suggested and we agree that:

We are far from a full understanding of why brain-based understandings are taken up enthusiastically in some institutions and not in others, and by some scholars and not others … The scholarly debates over what critical position to take in relation to neurological accounts of human social life would benefit from looking into what is at stake among non-experts struggling over how to position the brain in their lives (Martin, 2010).

## Stakeholder voices on neuroimaging

Different approaches have been used to date to explore how neuroimaging may affect mental health care now and in the future. For example, Joseph Dumit and Simon Cohn have used ethnographic approaches to explore the ways in which images of brain function influence researcher, patient, and popular understandings of mental illness and intervention (Dumit, 1999, 2003, 2004; Cohn, 2010). Dumit's 2004 ethnography *Picturing Personhood* pioneered inquiry into stakeholder perspectives on neuroimaging, integrating interviews with PET researchers, semiotic analyses, observation, and theoretical reflection into a coherent narrative about the emergence and influence of neuroimages. Although Dumit's work focused exclusively on PET scanning, it set the scientific, clinical, legal, and societal context for neuroimaging and provided a backdrop for future empirical work. Dumit, as well as Cohn, probed the ways in which neuroimages create new insights into and beliefs about the brain, personal identity, and lived experience. This line of inquiry has developed into the growing body of “critical neuroscience” literature, a discourse that examines how neuroscience generates new knowledge and the ways in which that knowledge is interpreted and incorporated into social and political interactions (Choudhury and Slaby, 2012).

Our own group has used an anticipatory ethics approach—the study of ethics and probable social issues associated with emerging technologies prior to mainstream use—with survey and interview methods to probe complementary questions about the potential of neuroimaging to impact patient, health care provider, and parent groups as well as the lived experience of mental health care (Illes et al., 2008; Borgelt et al., 2011, 2012a,b; Anderson and Illes, 2012; Buchman et al., 2012; Eijkholt et al., 2012; Anderson et al., under review). In one survey study, adults living with major depressive disorder and psychiatrists reported high receptivity to the use of neuroimaging for treatment planning (Illes et al., 2008). Their hope was that brain scans would help patients to understand and cope with the mental illness and mitigate the effects of stigma and self-blame. The data suggested that neuroimaging could play a significant role in alleviating the social burden attached to a diagnosis of mental illness. Later studies using qualitative methods enrich this initial work with evidence about beliefs about and hopes for what the technology would provide for diagnosis and treatment tailoring of mental health disorders, again across stakeholder groups of researchers (Cohn, 2010), providers (Borgelt et al., 2011, 2012a,b), adults with mood, psychotic, and obsessive compulsive disorders (Cohn, 2010; Buchman et al., 2012), and parents of children with ADHD (Borgelt et al., 2012b).

Key to the appeal for patients, parents, and even some providers is the apparent objectivity of neuroimages. Perceptions of objectivity create a new frame through which patients and parents construct an understanding of mental illness as brain disorder, with black-and-white evidence of neurobiological dysfunction replacing subjective feelings of “being crazy” (Dumit, 2003; Cohn, 2010; Buchman et al., 2012). Interviews of adults with mood disorder suggest that the visual reaffirmation offered by neuroimages legitimizes the experience of mental illness with authoritative representation of the phenomenon (Dumit, 2003; Cohn, 2010; Buchman et al., 2012). This representation leads many patients to adopt a dualist distinction between *me* and *my brain*, or *me* and *my illness*. Indeed, patients and parents hope that the stigmas so messily entangled in mental illness—dynamic webs of biology, psychology, spirituality, and sociality—will slip away from the glossy neuroimage. Patients wish to mitigate social distance and fear (Buchman et al., 2012), while parents strive to dispel what they perceive to be a common practice of parent-blame (Borgelt et al., 2012b).

In addition to reducing stigma and improving understanding of their child, parents also hang their high receptivity to neuroimaging on a desire for objective diagnostics to ameliorate doubts about medication and to aid their efforts to champion resources for their children (Borgelt et al., 2012b). For parents, technology is purely instrumental and its appeal may reduce to a simple, enduring hope for “*anything that would help*” (Borgelt et al., 2012b).

Adult patients and parents of children with ADHD voice few or no explicit concerns about the potential risks of neuroimaging (Cohn, 2010; Borgelt et al., 2012b; Buchman et al., 2012). However, they do underscore the vulnerability that corresponds to high hopes and expectations and thereby point to areas in which clinical and ethical challenges will likely arise. Inextricable from high hope is the potential for great disappointment. For example, while these stakeholders desire decreased social stigmatization, prior survey work reports that uptake of biological explanations for mental illness by the general public does not necessarily correlate to increased tolerance and may even reinforce certain stigmatizing practices such as social distancing (Phelan, 2005; Pescosolido et al., 2010; Angermeyer et al., 2011). Although the persuasive power of neuroimages may be positively applied to endeavors such as promoting patient and public education and informing treatment decision-making, caution and attention from the academic and medical communities are needed to mitigate patient manipulation or exploitation, inappropriate application for clinical diagnosis, and coercive uses for treatment compliance. Not surprisingly, providers are more cautious. They do describe downstream clinical benefits of correlating mental illness to neurobiological mechanisms, such as refining the categorization of disorders, improving diagnostic criteria, and enabling prediction of treatment response. Balancing their discussion of benefit, however, providers focus more significantly on the potential for compromised patient care, diminished value of the comprehensive assessment, and disruptions in doctor-patient communication (Illes et al., 2008). Their anticipation of the risks of predictive applications of neuroimaging are particularly salient.

Together with researchers, providers also diverge from patients and parents on the point that neuroimaging promises an objective representation of subjective experience with a “*picture of a broken brain*” (Buchman et al., 2012). They view neuroimaging as a statistically derived tool rather than a signifier of objective truth. As a by-product, researchers, and providers couch their discussion of potential risks and benefits associated with clinical neuroimaging in mental health care and are more cognizant (or at least more expressive) of the current limitations of the technology (Cohn, 2010; Borgelt et al., 2011).

**Figure 1:**
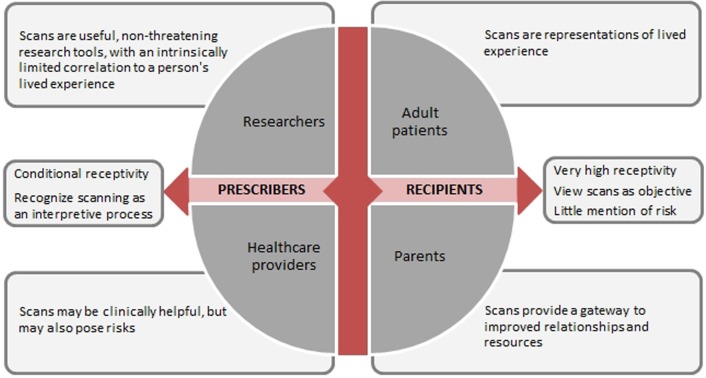
**Translational relationships to brain scan technology**.

Given the differences in perspectives among stakeholder groups regarding beliefs about the nature of neuroimaging and recognition of its limitations and risks, a key determinant of stakeholders' views is their relationship to the technology—that is, whether they are a recipient (patient or parent) or a prescriber (researcher or provider) of the technology (Figure 1). The impact of one's relationship to technology overarches other possible determinants such as diagnostic category or decision-making role. Though intuitive to some extent, identifying potential mediators of stakeholder perceptions and such as this can only be supported once stakeholder perspectives are solicited and compared.

## Moving forward: now what?

High receptivity to neuroimaging emerges across studies of stakeholder groups—among neuroimaging researchers and mental health care providers who see it as a valuable tool for unlocking the mechanisms of mental illness and improving patient care, and among adult patient and parent groups who hope that neuroimaging will help transform their lived experience. These are well-articulated messages despite differences in study design, in the milieu in which they were elucidated, in the nuances of qualitative analysis, and even with respect to the neuroimaging modality and format to which they refer (Beaulieu, 2002; Keehner et al., 2011). However, high receptivity to neuroimaging among these stakeholders does not alone justify a normative conclusion to usher neuroimaging faster along the translational pathway.

Indeed, this pertains to how the academic, medical, and policy communities should act on these data—a question that has been central in debates among academics seeking to integrate normative and empirical approaches to ethical and policy challenges (Borry et al., 2005; Goldenberg, 2005; Arras, 2007; Draper and Ives, 2007; Kon, 2009; Braddock and Magnus, 2010). Purely normative approaches to ethics and policy development may be weakened by unfounded assumptions and a disconnect between armchair rationale and the realities of context and culture. However, critics of empirical approaches caution against simple poll-taking or consensus-building because they see it narrowly describing what *is* rather than synthesizing what *ought* to be. Such criticisms might impugn a review of stakeholder perspectives, arguing that such data exacerbate the is-ought problem and lack normative directivity. Empirical work in anticipatory ethics—such as on future applications of neuroimaging in mental health care—is even a step removed from describing what *is* in its attempt to forecast the elusive *will be*.

We, like others (Borry et al., 2005; Draper and Ives, 2007), however, envision an evidence-based ethics that is attentive to the complex social, phenomenological, moral, and technological dimensions of issues in biomedicine, that is equally reflective about its limitations and is critically normative. While we acknowledge that the hypothetical nature of anticipatory research on stakeholder views poses inherent limitations on its conclusions, hypotheticals conversely serve as perhaps its greatest strength. Engaging the complexity of stakeholder views proactively, before neuroimaging is ushered further toward the doors of the mainstream mental health clinic, allows for upstream incorporation of the views they express as well as the upfront management of concerns or hype. And, to whom should policy-makers listen when perspectives diverge? To this question, the pragmatic answer is: all of them. The practical reality is that divergences of perspective across groups suggest probable tensions that, if left unaddressed, will inevitably play out in the clinic. Moving forward with empirical inquiry, normative analysis, and policy development—the “now what” question—we encourage continued efforts to collect stakeholder perspectives, integrate them upstream of clinical translation, and proactively address ethical and practical challenges borne out by differences in stakeholders' hopes, concerns, and changing conceptions of mental health.

## Conclusion

Past and contemporary research has reinforced the importance of the voice of stakeholders in the translational pathway for neuroimaging, and revealed how images of brain function will be received, perceived, and incorporated into the experience mental health care. Neuroimaging is expected to have a profound, expansive impact on the conceptualization of mental illness and provision of mental health care. The magnitude and nature of the effects of neuroimaging on its end-users and social context must continue to be elucidated to inform policy and practice in the future.

### Conflict of interest statement

The authors declare that the research was conducted in the absence of any commercial or financial relationships that could be construed as a potential conflict of interest.
